# Genetic variation and phylogeography of the magpie’s genus Pica in the Holarctic

**DOI:** 10.18699/vjgb-25-61

**Published:** 2025-07

**Authors:** A.P. Kryukov

**Affiliations:** Federal Scientific Center of the East Asia Terrestrial Biodiversity of the Far Eastern Branch of the Russian Academy of Sciences, Vladivostok, Russia

**Keywords:** mitochondrial DNA, Control Region, speciation, refugia, range, Pleistocene, митохондриальная ДНК, контрольный регион, видообразование, рефугиум, ареал, плейстоцен

## Abstract

The theory of Pleistocene refugia is often used to explain the population genetic structure of species. However, it does not fully account for the diversity of species-specific characteristics and natural conditions. The genus Pica, which is widespread in the Holarctic, provides an ideal model for studying phylogeographic patterns in order to better understand processes of diversification and speciation. Markers of mitochondrial DNA remain widely used in phylogeographic studies, despite advances of whole genome techniques. We have summarized published research on the mitochondrial DNA Control Region (CR) variation, based on data from 279 samples which represent the majority of extant taxa across the entire distribution range of the genus. In the phylogenetic trees and networks, we found several cases of reciprocal monophyly among most allopatric species and subspecies, and in addition some examples of paraphyly and polyphyly. Bayesian skyline plots were calculated to explore population dynamics over time. They showed varying longevity of the lineages since their origin or after experiencing a bottleneck, e. g., in the case of the Kamchatka population, as well as unequal rates of expansion. In most cases, speciation followed a geographic model involving expansion and vicariance, sometimes with divergence in refugia. Somewhere, peripatric speciation may have happened due to separation of a marginal populations. By comparing haplotype composition among populations, we traced the origin of the recently established populations on Hokkaido and Kyushu islands from a limited number of colonizers from the mainland. Isolated cases of species in statu nascendi were identified through evidence of incomplete lineage sorting, leading to paraphyly, or signs of limited unidirectional interspecies introgression of nuclear genes in secondary contact zones. Several hypotheses regarding the formation of the magpie´s range are proposed. Various evolutionary scenarios found in the genus Pica were compared to those reported for the other bird species in a number of literature sources.

## Abstract (ru)

Для объяснения истории формирования популяционно-генетической структуры видов часто привлекают теорию плейстоценовых рефугиумов. Однако она не может охватить все многообразие видоспецифических особенностей и природных ситуаций. Широко распространенный в Голарктике род сорок Pica оказался удобным для построения картины филогеографии с целью познания процессов диверсификации и видообразования. Маркеры митохондриальной ДНК по-прежнему широко используются в филогеографических исследованиях, несмотря на прогресс методов полногеномного секвенирования. Представлен обзор результатов анализа изменчивости контрольного региона (CR) митохондриальной ДНК по опубликованным нами данным от 279 образцов, представляющих подавляющее большинство таксонов сорок. На филогенетических деревьях и сетях гаплотипов мы обнаружили, помимо реципрокной монофилии аллопатрических видов и подвидов, примеры парафилии и полифилии. Контурные диаграммы демографии популяций показали разную продолжительность жизни линий после их основания либо прохождения «бутылочного горлышка», как в камчатской популяции, и неодинаковую интенсивность экспансий. Видообразование сорок проходило, вероятно, по географической модели за счет расселения и викарирования, в том числе с изоляцией и дивергенцией в рефугиумах. В ряде случаев предполагается перипатрическое видообразование за счет отделения краевых изолятов. По гаплотипическому составу молодых популяций островов Хоккайдо и Кюсю прослежены материковые источники их происхождения. Случаи незавершенного видообразования выявлены по наличию неполной сортировки линий, приводящей к парафилии, либо современной межвидовой интрогрессии ядерных генов. Предложены гипотезы формирования ареалов некоторых таксонов сорок. Привлечение большого объема литературы позволило сопоставить отмеченные в роде Pica разнообразные эволюционные сценарии с описанными для других видов птиц.

## Introduction

The current distribution and genetic structure of species are primarily shaped by processes which took place during the Quaternary (Avise, Walker, 1998; Hewitt, 2000). However, this perspective often underestimates overlapping processes, such as invasions, and shifts the species’ range boundaries, ecological and anthropogenic changes, population size fluctuations and secondary contacts with or without hybridization. Distinguishing the genetic consequences of these factors is crucial for a better understanding of diverse processes driving diversification and speciation. Widespread polytypic species – or complexes of closely related species – are of particular interest for building hypotheses of range formation and for learning the divergence mechanisms. Modern phylogeographic approaches have been adopted to address a wide range of evolutionary and genetic problems (Avise, 2000; Bannikova, 2004; Abramson, 2007; Zink, Barrowclough, 2008; Kholodova, 2009; Edwards et al., 2015, 2016a, b, 2022).

Phylogeographic studies of birds and other animals have made significant advances (reviews: Zink, 1996; Joseph, Omland, 2009; Hickerson et al., 2010; Toews, Brelsford, 2012; McCormack et al., 2013; Ottenburghs et al., 2019; Pârâu, Wink, 2021; Fu, Wen, 2023). Multilocus and genomic databases are expanding, analytic approaches and hypotheses testing methods are becoming more sophisticated, species distribution and ecological niches are being modeled, comparative as well as statistical phylogeography develops. Phylogeographic structures of many bird species, first European and American, were investigated, primarily by using traditional mitochondrial DNA markers. These structures and speciation ways are usually associated with the refugial phenomenon, in which recurring glaciation cycles forced populations to retreat southward and form isolated populations (Taberlet et al., 1998; Hewitt, 2000, 2004). Within such refugia, populations diverged due to genetic drift or/and local selection. In the case of long enough isolation over several glacial cycles, speciation could occur. Populations that underwent a bottleneck in a refugium, suffered a loss of diversity. On the other hand, fusion of the diverged populations within a refugium could increase variation.

However, refugial theory cannot fully explain all of the diverse cases found in nature. Range expansions occurred during the brief interglacials and especially after the Last Glacial Maximum (LGM), when advancing populations experienced new environmental conditions which could drive divergence. Consequently, the postglacial expansion hypothesis provides an alternative pathway of speciation within a short time frame, rather than through refugial isolation over a set of glacial cycles (Hansson et al., 2008). The case of exceptionally fast speciation was reported for the genus Junco, where five genetically distinct morphotypes of the species level evolved within ~10,000 years, during a single postglacial expansion (Mila et al., 2007). This challenges the idea that speciation occurred throughout the entire Pleistocene (Avise, Walker, 1998) or at least over the last 250 thousand years (Johnson, Cicero, 2004). Other reports suggest that the principal diversification and speciation events occurred as early as Pliocene and concluded in Pleistocene (Klicka, Zink, 1997). In some cases, phylogeographic breaks may appear within a continuous range, even without any geographic barriers to gene flow, particularly when individual dispersion distances are limited or population size diminished as was demonstrated for the greenish warbler Phylloscopus trochiloides (Irwin, 2002). In addition, zones of secondary contact and hybridization appeared in other cases of postglacial expansion. These processes are diverse, usually species-specific and insufficiently studied.

**Fig. 1.**
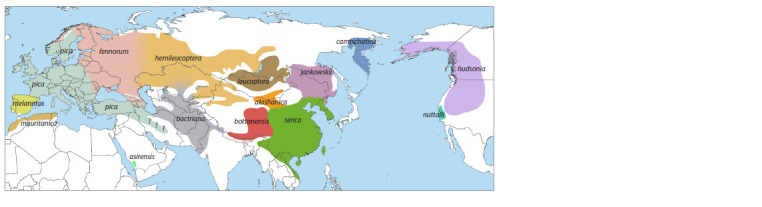
Distribution of magpies P. pica. From (Kryukov et al. 2022), with changes.

Despite being one of the best-known birds, “the magpie” keeps still many mysteries. Species of the genus Pica are widely distributed across the Holarctic from Western Europe to North America and from the arctic tundra to the Arabian deserts (Fig. 1). The genus includes forms with varying degrees of relatedness. Aside from the “good” allopatric species, several subspecies-level forms intergradate in Eurasia, while others form isolates. This naturally causes disputes over their taxonomic rank: whether they should be classified as separate species or subspecies. Interestingly, no magpie species are sympatric. For a long time, all magpies were classified as one species P. pica (Linnaeus, 1758) with 9–15 subspecies; however, this species was divided into several species after genetic approaches were adopted. Current taxonomic classifications accept seven species of magpies (Song et al., 2018; Madge et al., 2020; Gill et al., 2021), although the taxonomy of the genus remains a subject of debate.

Isolated populations of North Africa, Arabian Peninsula and Central China are accepted as distinct species based on analyses of mitochondrial and, to some extent, nuclear markers: Maghreb magpie (P. mauritanica Malherbe, 1845), Asir magpie (P. asirensis Bates, 1936), and black-rumped or Tibetan magpie (P. bottanensis Delessert, 1840), respectively (Song et al., 2018). Likewise, two allopatric forms of North America – black-billed magpie (P. hudsonia (Sabine, 1823)) and yellow-billed magpie (P. nuttalli (Audubon, 1837)) – are clearly distinct by phenotype and genetically diverse. This classification is further supported by reciprocal monophyly in the phylogenetic trees based on single genes (Song et al., 2018) and by complete mitochondrial genome analysis (Kryukov et al., 2020, 2024). The isolated Kamchatka population has been identified as a distinct lineage among Pica subspecies (Lee S. et al., 2003) and traditionally treated as subspecies P. pica camtschatica, but its potential elevation to species rank is a matter of discussion.

The known gap between the western and eastern subspecies groups in South Siberia has not been previously investigated genetically. They differ by both phenotype and calls (Ebels, 2003; Kryukov et al., 2017). Our analysis of the mt cyt b gene and CR estimated p-distances between them as 4–5 % (Kryukov et al., 2004, 2017; Haring et al., 2007). These findings became the background for separating the eastern magpie P. serica Gould, 1845 from the former single species P. pica (Song et al., 2018; Madge et al., 2020). Thus, the modern taxonomic scheme of the genus includes five monotypic species P. mauritanica, P. asirensis, P. bottanensis, P. hudsonia and P. nuttalli, along with the polytypic species P. pica comprising the subspecies P. p. pica (Linnaeus, 1758), P. p. fennorum Lönnberg, 1927, P. p. hemileucoptera Stegmann, 1928, P. p. bactriana Bonaparte, 1850, P. p. leucoptera Gould, 1862, P. p. melanotos A.E. Brehm, 1857 and P. p. camtschatica Stejneger, 1884; and P. serica including the subspecies P. s. serica Gould, 1845, P. s. jankowskii Stegmann, 1928 and P. s. alashanica Stegmann, 1928 (Winkler et al., 2020; Gill et al., 2021, with small corrections in subspecies).

The above-mentioned range gap between P. pica and P. serica deserves special attention. The gap was reported by the ornithologists as early as the last century (Stegmann, 1932; Rustamov, 1954), but was ignored in most major studies. The range of P. pica therefore appeared to stretch continuously from the Iberian Peninsula to the Sea of Okhotsk (Goodwin, 1986; del Hoyo, Collar, 2016). We established that the gap in fact exists and coincides with a discontinuity in mtDNA. However, it is gradually filling up before our eyes due to the range expansion of the eastern subspecies P. s. jankowskii westward along the Amur River valley and the Siberian subspecies P. p. leucoptera moving in the opposite direction (Goroshko et al., 2018). It was discovered that a few decades ago these populations came into contact and hybridization started. This zone was the subject of our recent integrative study (Kryukov et al., 2022). It revealed asymmetric introgression using nuclear single nucleotide polymorphism (SNP) analyses. Furthermore, a statistically significant decrease in breeding success was found in a hybrid population in Eastern Mongolia. This implies selection against hybrids and, consequently, limitation of introgression (Kryukov, 2019; Kryukov, Goroshko, 2025).

Despite extensive research on distribution, ecology and variability of magpies throughout the genus’ range, a comprehensive understanding of the relationships and origins of the taxa – and the genus in general – is still lacking. The aim of the current study is to summarize both our own and previously published data on genetic variation, phylogeography and population dynamics of (nearly) all Pica taxa and to propose a hypothesis on the formation of their ranges. As our main genetic marker, we used the mitochondrial Control Region (CR) which is well-known as a frequently used marker at lower taxonomic levels, widely applied in phylogeographic studies. This noncoding region is one of the most variable and phylogenetically informative regions of mtDNA (Baker, Marshall, 1997; Saunders, Edwards, 2000; Barker et al., 2012). In total, we obtained and analysed 279 sequences ranging in length from 1,298 to 1,310 nucleotide pairs from the samples representing almost all taxa of the genus Pica (Kryukov et al., 2004, 2017, 2022; Haring et al., 2007). The origin of the samples, museum numbers and GenBank accession numbers are presented in Table S11. In addition, fragments of mt CR of P. hudsonia and P. nuttalli were extracted from their total mitogenomes published by Kryukov et al. (2024). We applied commonly accepted methods to analyse nucleotide and haplotype variation, perform neutrality tests, model population dynamics, construct haplotype networks and phylogenetic trees, and estimate divergence times. These methodologies had been described in details in our studies mentioned above. Published data on both mitochondrial and nuclear genes were considered for the discussion of our findings.


Supplementary Materials are available in the online version of the paper: https://vavilov.elpub.ru/jour/manager/files/Suppl_Kryukov_Engl_29_4.pdf


## Origin of the magpie genus Pica

Origin of the genus Pica and the classification of its close relatives remain uncertain. Molecular phylogenetic reconstructions suggest that the ancestral forms of Corvidеs (previously referred to as “core Corvoidea”) diversified during a period of insular isolation as a result of creation of the proto- Papua archipelago after its separation from Australia in the late Eocene–Oligocene (Jønsson et al., 2011; Aggerbeck et al., 2014). Subsequently these birds spread across Asia and other continents. The family Corvidae is believed to have originated in Southeast Asia (Ericson et al., 2005). However, the phylogenetic position of genus Pica remains unresolved, and a range of studies indicate various closely related and sister genera. Proposed sister genera include Ptilostomus and Podoces based on sequences of one mitochondrial and two nuclear genes (Ericson et al., 2005); Zavattariornis based on the mitochondrial cytochrome b gene (Ekman, Ericson, 2006); Nucifraga and Perisoreus based on the mitochondrial Control Region (Haring et al., 2012); Podoces and Carrulus based on the complete mitogenome (Iqbal et al., 2020). However, no study has provided a comprehensive analysis of all possible related genera, leaving the evolutionary origins of the genus Pica speculative.

**Fig. 2.**
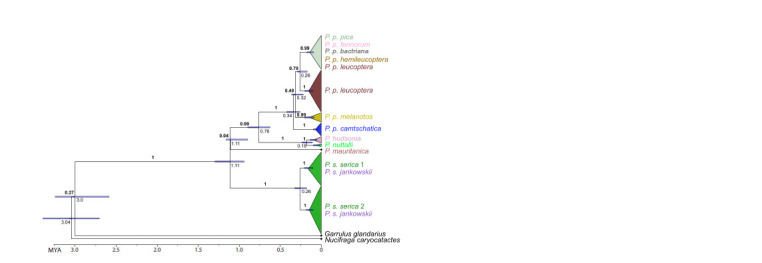
Time-calibrated Bayesian inference tree based on complete mitochondrial Control Region sequences of Pica species and outgroup Bold figures indicate Bayesian posterior probabilities. Values below the nodes indicate divergence time estimates (in millions of years), aligned with the time scale below. Blue bars next to the nodes indicate 95 % credibility intervals for age estimates. Triangle widths reflect specimen numbers. Colors of triangles and taxon names correspond to those at the map and network.

The basal split of the eastern magpie P. serica in the phylogenetic tree (Fig. 2) supports the hypothesis that the genus Pica originated in Southeast Asia. Its subsequent expansion across the continent was likely linked to the Holocene agricultural centers of south China and Mesopotamia (Nazarenko, 1982). However, our molecular dating suggests that the main divergence events within the genus occurred earlier, between 1 million years ago (hereafter Mya) and 200 thousand years ago (hereafter kya) (Fig. 2). There is a reason to believe that the evolution of magpies was clearly associated with grazing mammals. These animals provide a steady food source in the form of ectoparasites, as well as insects and other small animals disturbed while grazing. Some researchers even proposed a mutualistic relationship with ungulates (Londei, 2018). The long-stepped tail of magpies may have originally functioned as a balancer for perching on the backs of moving ungulates. The role in maneuverable flight among trees may have developed secondarily (Londei, 2018). Magpies primarily forage in short grass and likely spread mainly across grasslands and pastures. With the emergence and expansion of human populations, their high adaptability allowed them to occupy anthropogenic landscapes where they successfully reproduced. Magpies are mainly sedentary, but display a tendency to vagrancy including even hitchhiking on ships, as discussed below.

## Phylogeny of the magpies

**Fig. 3.**
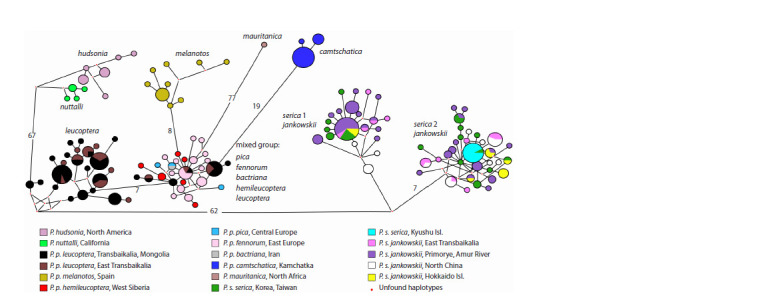
Phylogenetic Median Joining network based on complete mitochondrial Control Region sequences. Sizes of circles correspond to the number of birds sharing this haplotype; branch lengths are proportional to the number of substitutions and those over 6 are shown at the nodes.

Despite analyzing only a rather short part of each mitogenome, a high-resolution phylogenetic tree was obtained for all the principal branches, representing nearly all taxa of the genus Pica (Fig. 2). Deep divergence revealed between all main lineages generally corresponds to current taxonomic scheme at the species level (Fig. 2 and 3). The three main branches of the tree form a polytomy with deep divergence: 1) eastern magpie P. serica, 2) P. pica including its subspecies and the related P. hudsonia and P. nuttalli, and 3) the North-African P. mauritanica. The species P. mauritanica, P. hudsonia, and P. nuttalli along with the subspecies P. p. camtschatica and P. p. melanotos are reciprocally monophyletic. P. serica is monophyletic, but consists of two lineages: serica + jankowskii 1 and serica + jankowskii 2 (further briefly serica 1 and serica 2). Subspecies P. p. leucoptera is paraphyletic regarding the other subspecies of P. pica.

The highest nucleotide variation and number of pairwise differences were found in the leucoptera lineage, while the lowest occurred in the camtschatica lineage (Table 1). Haplotype variation is close in all lineages, with the exception of the lowered variation in camtschatica. Interspecies nucleotide substitution level was from 4 to 77 (1–6 % p-distance), while subspecies differences ranged from 0 to 19 substitutions (up to 2 %) (Fig. 3, Table 2). P. hudsonia and P. nuttalli where the closest related taxa. Some lineages comprised several taxa, such as the subspecies P. p. pica, P. p. fennorum, P. p. bactriana, P. p. hemileucoptera and P. p. leucoptera in one “mixed” group. In contrast, P. serica is represented by two highly distinct clades, each of them mixed regarding subspecies composition. A mutation rate of 0.025 substitutions/site/million years was used to calculate divergence times, consistent with the commonly applied substitution rate in CR of bird mtDNA (Freeland, Boag, 1999; Fok et al., 2002; Omland et al., 2006). Based on these calibrations, divergence of the main lineages of magpies took place in the mid-Pleistocene, about 1.1 Mya (Fig. 2), which is more recent than the previously proposed estimate of 2.5–3.1 Mya (Song et al., 2018). The most recent common ancestor (MRCA) of each lineage appeared in the late Pleistocene, approximately 66 kya or later (Table 1).

**Table 1.**
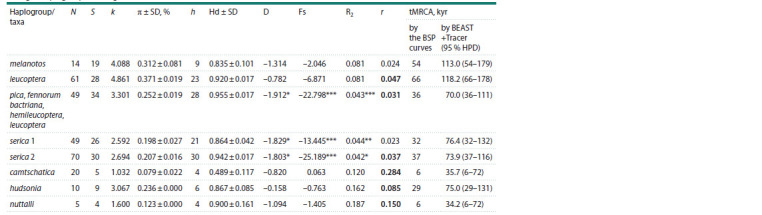
Parameters of variation, neutrality tests and times for the most recent common ancestor (tMRCA) estimations for eight haplogroups of the genus Pica Notе. N – sample size; S – number of polymorphic sites; k – average number of pairwise nucleotide differences; π ± SD – nucleotide diversity with standard deviation; h – number of haplotypes; Hd – haplotype diversity with standard deviation. Neutrality tests: D – Tajima’s; Fs – Fu’s; R2 – Ramos-Onsins & Rozas’s; their p-values: * p ≤ 0.05, ** p ≤ 0.01, *** p ≤ 0.001. r – Harpending’s raggedness index and its p-value, with insignificant values (p > 0.05) given in bold. tMRCA – times for the most recent common ancestor estimated by BSP plots and from Tracer, with 95 % HPD range, all in thousands of years.

**Table 2.**
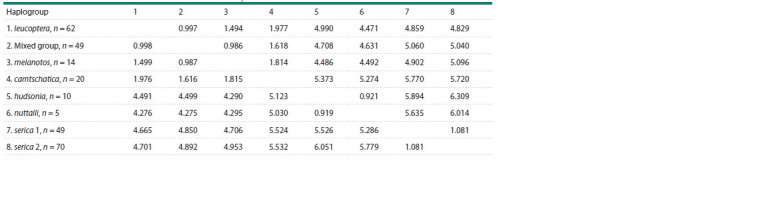
Average number of nucleotide substitutions per site (Dxy) and р-distances between Pica haplogroups (in %)

Deep divergence of the southeast lineage serica from the others was identified earlier by mitochondrial genes coding for 16S rRNA, tRNA-Leu and ND1 (Lee S. et al., 2003), as well as by cyt b (Kryukov et al., 2004), and was later confirmed by CR (Haring et al., 2007; Kryukov et al., 2017). At the same time, the position of the lineage of P. p. camtschatica remote from the eastern magpie was demonstrated (Lee S. et al., 2003). The pronounced divergence between the camtschatica lineage and the common hudsonia and nuttalli lineage (Fig. 2 and 3) contradicts the supposition of origin of American magpies form the Kamchatka subspecies (Lee S. at al., 2003). Instead, both American species seem to share a common ancestry with the south Siberian populations P. p. leucoptera. In contrast, the African P. mauritanica and the Iberian P. p. melanotos are more closely linked to the European-Siberian haplogroup (Fig. 3). The relationships among the remaining subspecies of P. pica are weakly resolved and appear as a polytomy in the tree (Fig. 2). However, based on a haplotype network and subspecies distribution, there is reason to assume that P. p. leucoptera may represent the ancestral haplogroup of all other subspecies of common magpie.

The subspecies P. p. leucoptera is paraphyletic regarding the subspecies group pica, fennorum, bactriana and hemileucoptera (Fig. 2 and 3). Species-level paraphyly is common in phylogenies based on animal mitochondrial genes and causes inconsistencies in taxon delimitation and discrepancies between gene and species phylogenies. A survey of 2,319 bird species revealed 23 % paraphyletic or polyphyletic for mtDNA (Funk, Omland, 2003). Mitochondrial paraphyly is distributed in 44 % of Australian bird species (Joseph, Omland, 2009). Misinterpretation of paraphyly may lead to false evolutionary inferences. There are numerous examples of erroneous taxonomy, and elevating subspecies status to species status can sometimes eliminate paraphyly. For examples, raising the rank of Corvus corax clarionensis to species status solved the problem of paraphyly in American ravens (McKay, Zink, 2010). During divergence from a common ancestor, lineages typically progress through phases of polyphyly, paraphyly, and ultimately reciprocal monophyly, driven by stochastic gene sorting (Avise, 2000). Therefore, a common cause of paraphyly is the incomplete lineage sorting due to recent speciation (Funk, Omland, 2003). In addition, introgressive hybridization, ancient or recent, may contribute to paraphyly, but distinguishing it from incomplete lineage sorting requires nuclear gene analysis involving coalescence models (Peters et al., 2007). In magpies, incomplete lineage sorting is the most likely explanation for subspecies-level paraphyly. This is supported by the observation that in early-stage divergence, common haplotypes are mostly in the centre of a clade, while taxon-specific haplotypes occupy the periphery (Omland et al., 2006). This pattern is clearly visible in the “mixed” haplogroup of the network (Fig. 3).

The presence of well-differentiated haplogroups is well confirmed by the networks we constructed. Each group corresponds to one or several taxa. The network constructed by the NeighborNet method with the SplitsTree software clearly shows the close affinity of P. hudsonia and P. nuttalli and the sister group relationship between serica 1 and serica 2 (Fig. S1). P. p. camtschatica appears to be most closely related to the “mixed” group. The Median Joining network provides a more detailed picture. Intergroup distances reach 77 substitutions (Fig. 3). The subspecies P. p. leucoptera is present in two haplogroups: in the “mixed” group and in the one including leucoptera only. The “mixed” group has a star-like structure with the single central haplotype shared by three subspecies. Notably, P. p. camtschatica is related to the mixed group, while both American species, P. hudsonia and P. nuttalli, are closer to the Siberian subspecies P. p. leucoptera. The groups serica 1 and serica 2 differ by 10 or more nucleotide substitutions, corresponding to a p-distance of 1.1 %. Representatives of the same populations from both subspecies P. s. serica and P. s. jankowskii were observed in each group without any apparent geographic pattern. The central haplotype of group serica 1 is shared by both subspecies (altogether from four populations), while in group serica 2, the main haplotype represents a sample from Korea. Thus, the phylogenetic tree and the haplotype networks show mutually complementary patterns offering insights into lineage divergence and ongoing evolutionary processes

## Population size dynamics

**Fig. 4.**
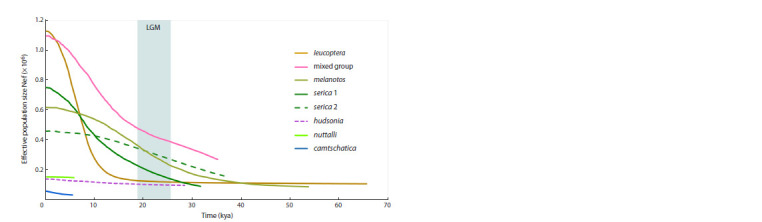
Bayesian skyline plots for population dynamics over time for eight haplogroups, based on the mitochondrial Control Region Curves of the skyline plot represent median values of effective female population size in millions (Nef ). Colored column depicts the LGM period.

The skyline plots based on Bayesian analysis of mitochondrial haplotypes reflect the dynamics of effective populations sizes for maternal populations and time since the most recent common ancestor (tMRCA). The earliest lineage emergence or bottleneck event was identified for the leucoptera population from Transbaikalia and Mongolia, while the latest was observed for nuttalli and camtschatica (Fig. 4). These two populations, together with hudsonia, exhibit relatively stable population sizes, while all the other populations show signs of population growth (Fig. 4). Among these, only three lineages (“mixed” and both serica lineages) showed significant support for population growth according to the three neutrality tests (Table 1). Yet the r-index suggests that population growth cannot be excluded for most lineages, except for melanotos and the serica 1 lineage. Pairwise nucleotide difference plots (not shown) displayed single leftwards peaks for all populations except melanotos, which does not contradict the hypothesis of population growth.

The diversity of contour diagram patterns depicting lineage or population dynamics suggests the following conclusions. The leucoptera lineage appears to have been formed earlier than the others, and the model predicts its most rapid growth after the LGM (Fig. 4). The “mixed” lineage, which has a star-like structure in the haplotype network, also underwent significant growth. Its growth started earlier than that of leucoptera and went in parallel with the expansion of the melanotos lineage. In eastern Eurasia, among the two P. serica lineages, the serica 1 lineage (represented by less samples) grew faster, corresponding to a star-like pattern with multiple representations of the common central haplotype (Fig. 3) and a shorter growth curve (Fig. 4). The recent growth of the “mixed” lineage as well as both of the serica lineages is supported by three neutrality tests (Table 1). The North American sister species P. hudsonia and P. nuttalli show population stability. P. nuttalli, which inhabits the extreme south of the American part of the genus range, diverged from common ancestor with P. hudsonia very recently. Short lifespan of the former species (Fig. 4) is supported by high haplotype and low nucleotide diversity (Table 1) which may indicate a founder effect. Short lifespan as well as low haplotype and nucleotide diversity is also observed in P. p. camtschatica (Fig. 4, Table 1), likely indicating a bottleneck rather than a founder effect. Generally, the pattern of population dynamics aligns with the estimated growth of the East Chinese clade after 100 kya (Zhang R. et al., 2012), as well as with the expansion of the east-Asian lineage around 60 kya, Eurasian lineage around 40 kya, and American lineage around 20 kya (Song et al., 2018).

## Phylogeography of magpies compared to other birds

The phylogeographic structure of species is primarily manifested in the presence of genetic clades (haplotype groups) that are distributed allopatrically or parapatrically and have been mainly identified by mtDNA data. For example, 14 species have been recorded in the Western Palearctic, which display clear distinctions between geographic lineages within species (Pârâu, Wink, 2021). These are mostly sedentary species. Three allopatric haplogroups corresponding to subspecies were revealed in the green woodpecker Picus viridis (Pons et al., 2011). In the middle spotted woodpecker Dendrocoptes medius, two groups were found, each associated with several separate refugia during the LGM (Kamp et al., 2019). Similar patterns have been observed in other species: three haplogroups in the Arctic warbler Phylloscopus borealis (Saitoh et al., 2010), three in the black-throated tit Aegithalos concinnus in east China (Dai et al., 2011), and three lineages with associated morphotypes in the Steller’s jay Cyanocitta stelleri (Cicero et al., 2022). Presence of three well-supported haplogroups, originated from three South-European Pleistocene refugia, was shown for the tawny owl Strix aluco (Brito, 2005). Five monophyletic groups with deep divergence in the early-middle Pleistocene and expansion before the LGM were discovered in the great tit Parus major (Zhao et al., 2012). The dipper Cinclus cinclus exhibits a complex structure with five lineages derived from two main refugia, Italian and Balkano- Karpatian, which were isolated during interglacials (Hourlay et al., 2008). In a number of examples, the presence of such divergent clades, often supported by additional distinct features as well, led to proposals for recognizing them as species. This applies to the horned lark Eremophila alpestris (Drovetski et al., 2014), the winter wren Troglodytes troglodytes (Toews, Irwin, 2008), the long-tailed rosefinch Carpodacus sibiricus (Liu et al., 2020) and the Arctic warbler Phylloscopus borealis (Alström et al., 2011). On the other hand, there are also cases where taxa were merged rather than split, for example, the lumping of three species of rosy-finches of the genus Leucosticte into a single species (Drovetski et al., 2009).

More common, however, is the lack of a clear genetic structuring of species across their ranges. For example, 90 % of the 145 analysed bird species of the western Palearctic show either a high degree of panmixia (46 species) or are only weakly differentiated throughout their ranges (85 species) (Pârâu, Wink, 2021). This finding was attributed to admixing of populations during both their retreat into southern refugia during glaciation and their subsequent post-glacial expansion. Overlapping haplogroup ranges were reported in several species, e. g., for the bunting Emberiza schoeniclus (Zink et al., 2008), the common rosefinch Carpodacus erythrinus (Pavlova et al., 2005) and the bearded vulture Gypaetus barbatus (Godoy et al., 2004). Four groups were identified in the long-tailed tit (Aegithalos caudatus complex), two of them occur allopatrically in southern China, while the other two, widespread across the northern Palearctic, overlap (Song et al., 2016). Similarly, among the four distinct clades of the wagtail Motacilla alba, three (N, SE and SW) partially overlap (Li X. et al., 2016). The European turtle dove Streptopelia turtur does not exhibit panmixia in the European part of its range. However, the three most frequent haplotypes were found in samples of all populations, from Greece to Spain and Great Britain (Calderon et al., 2016). These haplotypes differ by 2–6 substitutions only, which is a much smaller difference than that between the overlapping groups serica 1 and 2 (16 substitutions between the centers of the haplogroups in the network (Fig. 3)). Non-strict phylogeographic structure was revealed in the stonechat Saxicola torquata complex, which consists of three highly diverged and partially overlapping clades in the Palearctic (Zink et al., 2009). In the Chinese hwamei Leucodioptron canorum, three clades partially overlap in east China, with intensive gene flow between offspring of different refugia maintaining a high effective population size (Li S.H. et al., 2009). Similarly, in the vinous-throated parrotbill Paradoxornis webbianus, two lineages partly overlap as result of recent gene flow (Qu et al., 2012). In the common raven Corvus corax, two groups with divergence level of 4 % overlap in the western United States; this secondary contact allows for frequent interbreeding (Webb et al., 2011).

The examples of species exhibiting wide gene flow includes the hoopoe Upupa epops in Europe (Wang et al., 2017), the willow tit Parus montanus (Kvist et al., 2001; Pavlova et al., 2006) and the common sandpiper Actitis hypoleucos (Zink et al., 2008). Panmixia has been observed in the great spotted woodpecker Dendrocopos major throughout the central Palearctic (Perktaş, Quintero, 2013), and in the marsh warbler Acrocephalus palustris throughout Europe (Arbabi et al., 2014). In most of the examples cited, bottlenecking or expansion from a single refugium occurred. The genetic affinity among western magpie subspecies, except for the Iberian subspecies, can be explained by recent gene flow between populations (Fig. 2 and 3, Fig. S1).

The significant phylogeographic break in the magpie’s range was discovered in South Siberia (Kryukov et al., 2004), which led to the separation of the eastern magpie (P. serica) as a distinct species from the previously single species P. pica sensu lato. Such a division between closely related western and eastern taxa is observed rather often in species with wide trans-Palearctic ranges and is often reflected in clear mtDNA divergence. Examples of this East/West pattern include: the azure-winged magpie Cyanopica cyanus (Fok et al., 2002; Kryukov et al., 2004), the rook Corvus frugilegus (Haring et al., 2007; Salinas et al., 2021), the flycatcher Ficedula parva and the skylark Alauda arvensis (Zink et al., 2008). Apart from birds, similar breaks have been observed in other animals, e. g., in the narrow-headed vole Microtus gregalis (Abramson et al., 2006), the Siberian newt Salamandrella keyserlingii (Berman et al., 2005) and the wasp spider Argiope bruennichi (Krehenwinkel et al., 2016). Interestingly, localization of these phylogeographic breaks rarely coincides across species. Complex structure with division into a western clade (Europe and Caucasus) and an eastern clade (central, eastern Asia and Sino-Himalayas) was found in the Eurasian wren Nannus troglodytes (Albrecht et al., 2020). In the carrion crow Corvus corone and the hooded crow C. cornix, the divergence between mtDNA clades does not coincide with subdivision into subspecies (Kryukov, Suzuki, 2000; Haring et al., 2007). In other examples, subspecific division coincides with phylogeographic breaks: the two subspecies of the black kite Milvus migrans show clear mtDNA divergence, with a broad zone of intergradation in Siberia (Andreyenkova et al., 2021). At the same time, other widely distributed species have no such phylogeographic breaks, which may indicate their more recent evolutionary history or/and current gene flow.

## History of the formation of the magpie range

The evolutionary history of the genus Pica throughout its vast trans-Holarctic range has undergone multiple stages, and it seems difficult to completely reconstruct it. However, several key points may be identified. Vicariance, as a result of fragmentation of previously extensive ranges, together with local adaptations appears to be the main mechanism of speciation. Marginal populations might have diversified from isolated border population, in accordance with the peripatric speciation model, a variation of the geographic speciation model (Mayr, 1963). This hypothesis was discussed in details for the endemic species P. nuttalli in a study analyzing complete mitogenomes of magpies (Kryukov et al., 2024). A similar process may have led to the diversification of the isolated, small-range taxa, e. g., P. mauritanica, P. asirensis and P. p. camtschatica. The Asir magpie P. asirensis represents a remote isolate and is still barely studied genetically (Song et al., 2018). However, a recent study of two mitochondrial genes revealed its sister group relation to the Tibetan magpie P. bottanensis (Song et al., 2018). This observation suggests a hypothetical spreading of magpies along the belt of steppes and semideserts from East Asia to Arabia and further into North Africa in multiple waves, driven by repeated cooling and warming periods. Range fragmentation may have led to the formation of isolates such as the Asir and Maghreb magpies, which are identified as the most basal lineages according to mitochondrial phylogenetic reconstructions (Song et al., 2018). It is unlikely that the magpie carried on from North Africa to Europe since the Strait of Gibraltar separated them at the early Pliocene, ~5 Mya (Krijgsman, 2002). According to our data, the Maghreb and Iberian magpies are deeply diverged in both phenotypes and mtDNA, and the former species may represent a dead end to that hypothetical route. This scenario, which involves a few remote extant isolates, implies the past elimination of intermediate forms

**Fig. 5.**
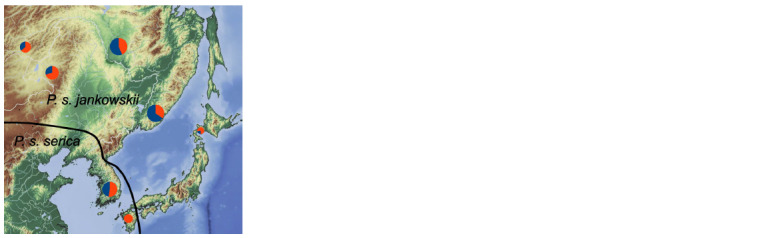
Genotypic content of the population of the eastern magpie P. serica. Proportion of serica 1 haplogroup representatives is shown in blue, and that of serica 2 in red.

The eastern magpie P. serica diverged from the other lineages in the middle Pleistocene (Fig. 2). The subspecies P. s. serica and P. s. jankowskii are geographically separated (Fig. 1). They are similar by plumage coloration, but clearly distinct by size and proportions (Red’kin et al., 2021). The finding of two haplogroups with genetic divergence at p- distance of 1.1 % within this species was unexpected (Fig. 2 and 3). Notably, both haplogroups serica 1 and serica 2 coexist in populations across the species’ entire range, from Eastern Transbaikalia in the west to Korea and Hokkaido in the east (Fig. 5). The proportions of both haplogroups vary between population, but not significantly. The only exception is the homogenic population of Kyushu Island, discussed below.

A similar sympatric pattern was reported for the red-backed shrike Lanius collurio, where two clearly diverged haplogroups with a genetic distance of 2.8 % coexist throughout Europe (Pârâu et al., 2019). A further example is the common redstart Phoenicurus phoenicurus: two haplogroups diverged by 5.1 % are sympatric in the whole of Western Europe (Hogner et al., 2012). In both cases, the most likely explanation of this rare phenomenon is the impact of recurrent glacial cycles throughout the Pleistocene. Populations retreated to the south in cooling periods and intermixed in refugia, e. g. in Iberia and the Balkans, while during the warming cycles they spread northward with repeated mixing. A similar pattern became evident in the European bee-eater Merops apiaster, where two star-like haplogroups, differing by only one substitution, connect haplogroups of populations from South Africa to Western Europe and China, illustrating the panmixia resulting from lineage mixing both after refugial isolation as well as in present times (Moura et al., 2019). A different case was observed in the great reed warbler Acrocephalus arundinaceus in Europe: two mitochondrial clades diverged 65–87 kya and partly overlapped on a wide range (Hansson et al., 2008). Presumably, they originated independently in two refugia located in Southern Europe and Middle East, respectively. The first expansion wave may have resulted in the occupation of the total range, while the second one was limited to its southern part only (Hansson et al., 2008). These clades are not isolated reproductively, because the length of isolation within refugia proved not to be long enough for establishing complete isolation barriers. It supports the hypothesis of postglacial expansion as a reason for speciation in a rather short period of time. Some other examples were listed above at the section Phylogeography.

Origination and establishment of the haplogroups serica 1 and serica 2 in the studied parts of Asia likely occurred in two refugia, which is supported by the post-Pleistocene divergence times and tMRCA estimates (Fig. 2, Table 1). While the southern part of the P. serica range was not surveyed by us, previous research on two nuclear genes did not reveal any genetic structure in Eastern China (Zhang R. et al., 2012). This suggests a spread from a single refugium and subsequent gene flow between populations. The star-like haplotype structures and the results of neutrality tests, for both haplogroups serica 1 and serica 2, do not contradict the hypothesis of population expansion, which might have started even before the LGM (Fig. 3 and 4, Table 1). A similar pattern has been observed in the great tit Parus major, where a clade in Eastern Asia started a wide expansion ~50 kya showing no impact from the LGM (Song et al., 2020).

In general, the region of eastern temperate Asia might have included many large and small refugia, not necessary the same for different species, in contrast to Europe, where the main refugia were identified in Iberia, the Apennines and the Balkans (Hewitt, 1996; Fu, Wen, 2023). Several species in Far East Asia demonstrate a deep divergence between haplogroups, from the Korean peninsula, on one side, and North-Eastern China and the Primorsky region in the Russian Far East, on the other. Such a pattern was found in the Siberian chipmunk Tamias sibiricus with a divergence level of 11 % (Lee M.Y. et al., 2008), the Asian wood mouse Apodemus peninsulae (Serizawa et al., 2002; Kim H.R., Park, 2015; Chelomina et al., 2024), the Korean field mouse Apodemus agrarius (Sakka et al., 2010), the tree frogs of the group Hyla japonica (Dufresnes et al., 2016), the Asiatic toad Bufo gargarizans (Borzée et al., 2017) and partly in the raccoon dog Nyctereutes procyonoides (Kim S.-I. et al., 2013). Refugia in both Eastern China and Korea were recognized for the black-spotted frog Pelophylax nigromaculata, with two lineages having diverged by 7.7 % (Zhang H. et al., 2008). Two refugia were also proposed for the bamboo partridge Bambusicola thoracica in China (Huang et al., 2010). Climatic oscillations having occurred in that region did not lead to total glaciation, and average temperatures decreased in the Korean peninsula by only 5–6 °C (Yi, Kim, 2010). Many species survived the LGM in their refugia and subsequently expanded northward, but not exclusively (Fu, Wen, 2023).

The refugial hypothesis in magpies and specifically those of two refugia in the east easily explains the existence of two rather deeply genetically diverged haplogroups: serica 1 and serica 2. One refugium may have been located in the Korean Peninsula, the most known refugium for Eastern Asia, as the central haplotype of group serica 2 in the network originated from Korea (Fig. 3). The ancestral population of group serica 1 likely has formed later and expanded faster (Fig. 4). The central haplotype of group serica 1 is widely distributed from Hokkaido to Transbaikalia, suggesting that its corresponding refugium might have been located in Primorye or Manchuria, similar to those described above for other species. The divergence level of these groups of 1.1 % (Table 2) is lower than in the examples presented above. This low level of divergence is likely due to a rather short period of isolation in their respective refugia with insufficient time for establishing reproductive isolation in case of secondary contact. In Northeast China, the coldest period of the Pleistocene was not the LGM, as in Europe and America, but the Dali glaciation (corresponding to Würm in Europe), which started 54–44 kya (Li J.J. et al., 2004; Zhang H. et al., 2008), when the refugia could have formed. This dating is close to our estimate of the post-glacial expansion of group serica 2 from the Korean Peninsula (37 kya), and a more recent and rapid expansion of serica 1 from Primorye (32 kya) (Table 1, Fig. 4). An alternative hypothesis could be that the two haplogroups originated from the same population as a result of ecological speciation. Yet such an explanation appears unlikely. First, it would require the existence of different ecological preferences; however, magpies are eurybionts. Second, it would contradict the differentiation of the two subspecies in their geographic ranges.

After merging, both lineages continued to spread without undergoing lineage sorting (Fig. 5). The population introduced to Kyushu is the only homogenic population among group serica 2 (Fig. 3), as explained below. The later and currently ongoing expansion westward along the Amur River valley and eastward to Hokkaido originated from a common population carrying both haplogroups. Overall, the formation of the current genetic structure within the range of P. serica implies a process of vicariant divergence in refugia, followed by expansion and subsequent admixing of representatives of both groups

Magpies are generally sedentary birds but exhibit nomadic tendencies. The initial diversification of the ancient lineage occurred before 1 Mya (Fig. 2), presumably during a period of expansion. This expansion from the original range of P. pica in Southeast Asia could have taken two routes: a southern path, south of the deserts and mountains of Central Asia to Arabia and then up to Northern Africa, and a northern path through South Siberia continuing westward. The southern route might have left relic populations, such as ancestors for the Asir and Maghreb magpies. Establishment of the modern populations completed much later, in the late Pleistocene. During the most recent interglacial since ~126 kya, South Siberian forests were replaced by steppe, albeit some forests and forest-steppe landscapes still persisted even during the maximal glaciations (Nazarenko, 1982; Granoszewski et al., 2005; Allen et al., 2010). The presence of sufficient herbaceous vegetation supported ungulates such as the saiga, which may have facilitated the spread of magpies. As magpies spread along the northern path, partial lineage sorting may have led to the formation of two haplogroups in South Siberia (diverged by 1 %) (Table 2). One of them (leucoptera) emerged around 66 kya, after the cold period of Marine Isotope Stage (MIS) 4 (71–57 kya). Over time it accumulated considerable nucleotide and haplotype diversity (Table 1). This lineage kept a stable population size until the LGM, after which fast numeric and range growth (Fig. 4) accompanied its wide distribution throughout Siberia and adjacent regions. Descendants of this lineage apparently migrated to Alaska across the Bering Strait and gave rise to the two American species P. hudsonia and P. nuttalli. The other (“mixed”) group might have been formed later, about 36 kya during a relatively warm period MIS 3 (Fig. 4). This may have occurred in the Altai-Sayan refugium (Pavelková Řičánková et al., 2014), also named the “center of spread” (de Lattin, 1957), or in the Hentei subcenter (Nazarenko, 1982). The star-like pattern in the haplotype network (Fig. 3) suggests that it underwent a bottleneck stage, followed by a rapid population growth, surpassing that of its sister lineage (Fig. 4). While spreading to the West, this lineage gave rise to a series of subspecies, ranging from P. p. hemileucoptera to the nominate P. p. pica (Fig. 1). The lack of lineage sorting in this process resulted in paraphyly of P. p. leucoptera (Fig. 2 and 3). In this line of subspecies, clinal variation in size and coloration follows an “isolation by distance” pattern (Cramp, Perrins, 1994), yet the close genetic affinity among these subspecies is indisputable (Fig. 3, Table 2). Physical barriers, such as the Ural Mountains, evidently do not prevent the gene flow between them. The subspecies P. p. melanotos presumably originated from this same “mixed” group but underwent deep divergence beyond the Pyrenees, as is discussed below. Additionally, the “mixed” group lineage gave rise to the Kamchatka subspecies, independently form the American lineage.

## Forming the populations of the islands and peninsulas

The homogenic population of Kyushu Island originated from a small number of birds introduced by people from Korea about 400 years ago (Eguchi, Kubo, 1992). It is likely, that just by chance, among the few founders of the population there were no representatives of the other haplogroup, namely serica 1. The population has been long protected and had a very restricted range, and only in the last 40 years it started to spread to the north of the island (Eguchi, 2016). The extreme genetic homogeneity of the Kyushu population illustrates the founder effect. Its origin is confirmed by a haplotype from Korea, which is identical to that of all birds from Kyushu (Fig. 3), as well as by analysis of six microsatellite loci (Mori et al., 2014).

The same study demonstrated that the Hokkaido Island population originated from Primorye or Korea rather than from Kyushu. Hokkaido and Primorye populations are closest in allele composition. In addition, mitochondrial haplotypes found in Hokkaido are as diverse as those from the presumably parental population of Primorye, and both haplogroups are present (Fig. 3). This indicates a fairly large number of founders of the Hokkaido population. The first nesting pairs of magpies were met in the port cities of south-western Hokkaido since 1993 (Horimoto, 2004), and the population has since grown to over 200 pairs (O. Hasegawa, personal com.). Notably, magpies do not breed on the neighboring islands of Sakhalin and Honshu. Their wings are not adapted for long-distance flights across open water. The most likely way of their arrival at Hokkaido is the occasional invasion with logging and other ships in the 1980–1990s, when cargo traffic between Primorye and Hokkaido was common. Observations of ornithologists suggest that magpies are attracted to ships in harbors as overnight roosting sites (Kryukov et al., 2017). It is likely that magpies use the same way to make it to Australia (GWA, 2017), Mauritius (Reinegger, Bhanda, 2024), and the eastern USA (Ebels, 2003). The same holds for the widely introduced house crows Corvus splendens (Ryall, 2016).

The Kamchatka magpie population shows a close affinity to the western forms and, according to genetic data and morphology, by means of migration from Siberia, and not from the southern P. serica. Magpies may have inhabited Kamchatka as early as the Pleistocene. During much of the last stage of the ice age, at least 40 % of the peninsula was covered by ice (Kamchatka…, 1974). However, magpies could have survived the harshest period in refugia of tree and bush vegetation in the Central Kamchatka depression. Postglacial expansion to the north and beyond the peninsula may have been influenced by human activity, including the development of settlements and reindeer herding. So far, the magpie penetrated the anthropogenic landscape of Kamchatka poorly, and in cities it only inhabits parks. The population’s extremely low nucleotide and haplotype diversity (Table 1) and the very short curve of the skyline plot (Fig. 4) indicate that it underwent a severe bottleneck in the recent past.

The Iberian Peninsula is recognized as one of the main European refugia alongside the Apennines and the Balkans (Hewitt, 1996). The magpie population in Iberia probably originated from the North, but became isolated behind the Pyrenees during glacial advances, leading to divergence within the refugium. Magpies have since occupied almost the entire peninsula and a recent population growth was noted, which fits our genetic analyses (Fig. 4). Haplotype diversity in P. p. melanotos is comparable to that of other widely distributed lineages (Table 1) suggesting the existence of not one but several refugia within the Iberian Peninsula. This aligns with the “refugium within refugium” concept (Gómez, Lunt, 2007; Abellán, Svenning, 2014). Genetic evidence for multiple refugia within Iberia has been reported for other species, e. g., the red-legged partridge Alectoris rufa (Ferrero et al., 2011), the ocellated lizard Lacerta lepida (Miraldo et al., 2011), and some fish and amphibian species (Gómez, Lunt, 2007). In no less than seven cases, the refugial ranges coincide across different species (Hewitt, 2011). We found no shared haplotypes between P. p. melanotos and the nominate subspecies P. p. pica, thus there is no clear evidence of gene flow beyond the peninsula. Nevertheless, this cannot be ruled out due to a lack of specimens from the Pyrenees, where intermediate melanotos × pica phenotypes have been reported (Martínez, 2016). On the other hand, in the Pyrenees there are contacts between the ranges of several animal and plant species (Hewitt, 2011; Poschel et al., 2018; Pons et al., 2019), which allows for the attribution of this ridge to “suture” hybrid zones (Remington, 1968). Unlike magpies, the Iberian rook population (Corvus frugilegus) has contributed to the northern populations, although it retains its genetic distinctness as shown by mtDNA and microsatellite analyses (Salinas et al., 2021). Generally, the Pyrenees appear to act as a barrier primarily for sedentary bird species (Neto et al., 2012), and for a few low-mobility amphibians and reptiles.

## Conclusion

The magpie genus Pica is of great interest for phylogeographic research due to its wide Holarctic distribution and notable phenotypic diversity. Despite its broad range, the genetic variation of magpies remains insufficiently investigated, particularly concerning nuclear genes. At the same time, exploring the highly variable, non-coding Control Region of mtDNA has proven effective for population genetic studies, revealing significant phylogeographic breaks among major genetic lineages. Interestingly, these breaks are not always consistent with the current taxonomic classification of the genus. The degree of reproductive isolation and divergence among genetic lineages in magpies varies considerably, from strict isolation in allopatry (all but one pair of species) to secondary contact with limited gene flow and selection against hybridization, as is the case in P. pica × P. serica. In contrast, other lineages that presumably diverged in separate refugia, such as serica 1 and serica 2, have completely merged. Also, the degree of reproductive isolation is weakly correlated with the level of mtDNA divergence. Despite substantial genetic differences, P. pica and P. serica interbreed rather successfully, and conversely, the parapatric species P. hudsonia and P. nuttalli, which are closely related in mtDNA, are fully reproductively isolated.

The speciation of magpies appears to have predominantly followed an allopatric model driven by dispersal and vicariance, including separation of marginal isolates (peripatric speciation). The hypothesis combining divergence in two refugia with partial lineage sorting and present gene flow between western subspecies is proposed to explain the paraphyly in the white-winged magpie P. p. leucoptera. The presence of two genetically distinct sympatric haplogroups serica 1 and serica 2 in the eastern magpie P. serica might be similarly explained by the hypothesis of divergence in refugia followed by mutual introgression and current expansion. The ongoing process of speciation is also evident in Transbaikalia and Mongolia, where incomplete reproductive isolation of P. pica and P. serica leads to limited asymmetric introgression of nuclear genes. The gene pool of the young insular populations of Kyushu and Hokkaido reflects the genetic makeup of their parental populations, while the Kamchatka population presumably experienced a glaciation and underwent a bottleneck. Both historical processes and current dynamics of species ranges shape the phylogeographic structure of magpies. Notably, all these phenomena can be detected by rather conventional analysis of the mtDNA Control Region. In total, a widespread and trivial taxon – the common magpie – presents us with a uniquely variable set of microevolutionary processes and their outcomes

## Conflict of interest

The authors declare no conflict of interest.
